# Layer-dependent semiconductor-metal transition of SnO/Si(001) heterostructure and device application

**DOI:** 10.1038/s41598-017-02832-8

**Published:** 2017-05-31

**Authors:** Chengcheng Xiao, Fang Wang, Yao Wang, Shengyuan A. Yang, Jianzhong Jiang, Ming Yang, Yunhao Lu, Shijie Wang, Yuanping Feng

**Affiliations:** 1 State Key Laboratory of Silicon Materials, School of Materials Science and Engineering, Zhejiang University, Hangzhou, 310027 China; 2 Research Laboratory for Quantum Materials, Singapore University of Technology and Design, Singapore, 487372 Singapore; 3 Institute of Materials Research and Engineering, Agency for Science, Technology and Research (A*-STAR), 2 Fusionopolis Way, Singapore, 138634 Singapore; 4 Department of Physics, National University of Singapore, Singapore, 117542 Singapore

## Abstract

As the downscaling of electronic devices continues, the problems of leakage currents and heat dissipation become more and more serious. To address these issues, new materials and new structures are explored. Here, we propose an interesting heterostructure made of ultrathin SnO layers on Si(001) surface. Our first-principle calculations show that a single layer of SnO on Si(001) surface is a semiconductor, but a bilayer SnO on the same surface is metallic. This metal-semiconductor dichotomy allows construction of single-2D-material-based electronic devices with low contact resistance and low leakage currents. In particular, due to the interaction between Sn and the Si substrate, the semiconducting monolayer-SnO/Si(001) has a highly anisotropic band structure with a much lighter hole effective mass along one direction than that of Si and most other 2D materials, indicating a high carrier mobility. Furthermore, by combining density functional theory and nonequilibrium Green’s function method, we directly investigate the transport characteristics of a field effect transistor based on the proposed heterostructures, which shows very low contact resistance, negligible leakage current, and easy gate control at a compact channel length.

## Introduction

The current electronic industry has been mainly based on semiconductors and Silicon has been the key enabler. As the downscaling of electronic devices continues, the length scale of field effect transistors (FETs) will soon be reduced to only several nanometers, and may eventually reach the level of single molecule or single atom. As a matter of fact, single-atom FETs have already been demonstrated^1, 2^. A number of issues need to be addressed for devices operating at this small scale. One of them is the leakage current due to quantum tunneling which becomes more and more serious when the device gets smaller. Another issue is the increased heat dissipation per unit area due to the contact resistance, when integrating a huge number of devices in a single wafer. These issues hinder the further increase of device performance and pose great challenges on the scaling guided by the Moore’s law^3^.

Various approaches have been proposed to overcome these problems^4, 5^. A possible solution is to adopt new materials/structures that can have compact size, high mobility, and low contact resistance. The rapid development in two-dimensional (2D) materials opens up unprecedented possibilities to realize novel functional devices^6^. As the thickness of 2D materials is only of a few atomic layers, devices based on such materials would have the advantages of compact sizes and easy integration. Graphene, the first 2D material produced experimentally, has many attractive properties such as ultrahigh carrier mobility. However, its zero bandgap is not suitable for logical devices. Until now, several semiconducting 2D materials have been experimentally realized. Among them, MoS_2_ has a suitable bandgap for electronic device applications. A FET based on single layer MoS_2_ has been demonstrated^7^ and its integrated circuits have been manufactured in the laboratory^8^. Other members in this transition metal dichalcogenide (TMDC) family have also been investigated. However, their carrier mobilities are not competitive with those of silicon and other conventional semiconductors^4^. Moreover, most of the 2D materials are intrinsically of n-type and p-type 2D materials are rare, which hinders the realization of p-n junctions based on the 2D materials.

Another challenge faced by 2D-material-based devices is to minimize the contact resistance. In electronic devices, conventional metals are typically used for electrodes which have different crystalline structures compared to the 2D materials. Due to the structural mismatch, large contact resistance is often found at the interface, which leads to heat dissipation. A possible solution to reduce the contact resistance is to replace the conventional metal electrode by a 2D metallic material that has similar lattice structure as the channel material. This concept was demonstrated by Kappera *et al*. using a single-layer MoS_2_-based transistor which consists of a semiconducting 2H-MoS_2_ channel connected to metallic 1T-MoS_2_^9^. However, the 1T phase of MoS_2_ is metastable and transforms to the 2H phase at room temperature which limits the operating temperature of the device. In another theoretical work, a FET based on ultra-thin PdS_2_ films was proposed, which utilizes a layer-dependent metal-semiconductor transition property of the material to minimize the contact resistance^10^. However, an external pressure is needed to make the PdS_2_ electrode metallic. Furthermore, the high cost of palladium would limit the large-scale application of the material. Therefore, a 2D material suitable for high performance logical devices remains to be discovered.

Recently, 2D SnO, with thickness of a few atomic layers, has been experimentally realized^11^. Notably, it exhibits an intrinsic p-type property. This rare property among the 2D materials makes SnO a promising component to realize 2D logical devices. Here, we perform first-principles calculations to investigate the structure and electronic properties of ultrathin SnO layers on Si substrate, to explore such an opportunity. We find that the interaction between SnO layer and the Si substrate strongly modifies the electronic properties of SnO. Compared to suspended SnO, the bandgap of SnO on Si substrate is decreased, and more importantly, the hole effective mass becomes much smaller along one direction and very anisotropic (~0.1 m_*e*_ along x direction and >15 m_*e*_ along y direction where m_*e*_ is the free electron mass). In addition, a semiconductor-to-semimetal transition takes place when the thickness of SnO increases from monolayer to bilayer. This makes it possible to build all-SnO FET device, with a monolayer SnO channel and bilayer/multilayer SnO electrode, on Si substrate. We further study the transport properties of such a device in detail, using first-principles methods and the non-equilibrium Green’s function (NEGF) technique. We demonstrate excellent performance of the device, including low contact resistance, negligible leakage current, and easy gate control. The present work thus not only reveals the interesting properties of the 2D-SnO/Si structure, but also presents a promising system for high performance next-generation electronic devices.

## Results and Discussion

**Figure 1:**
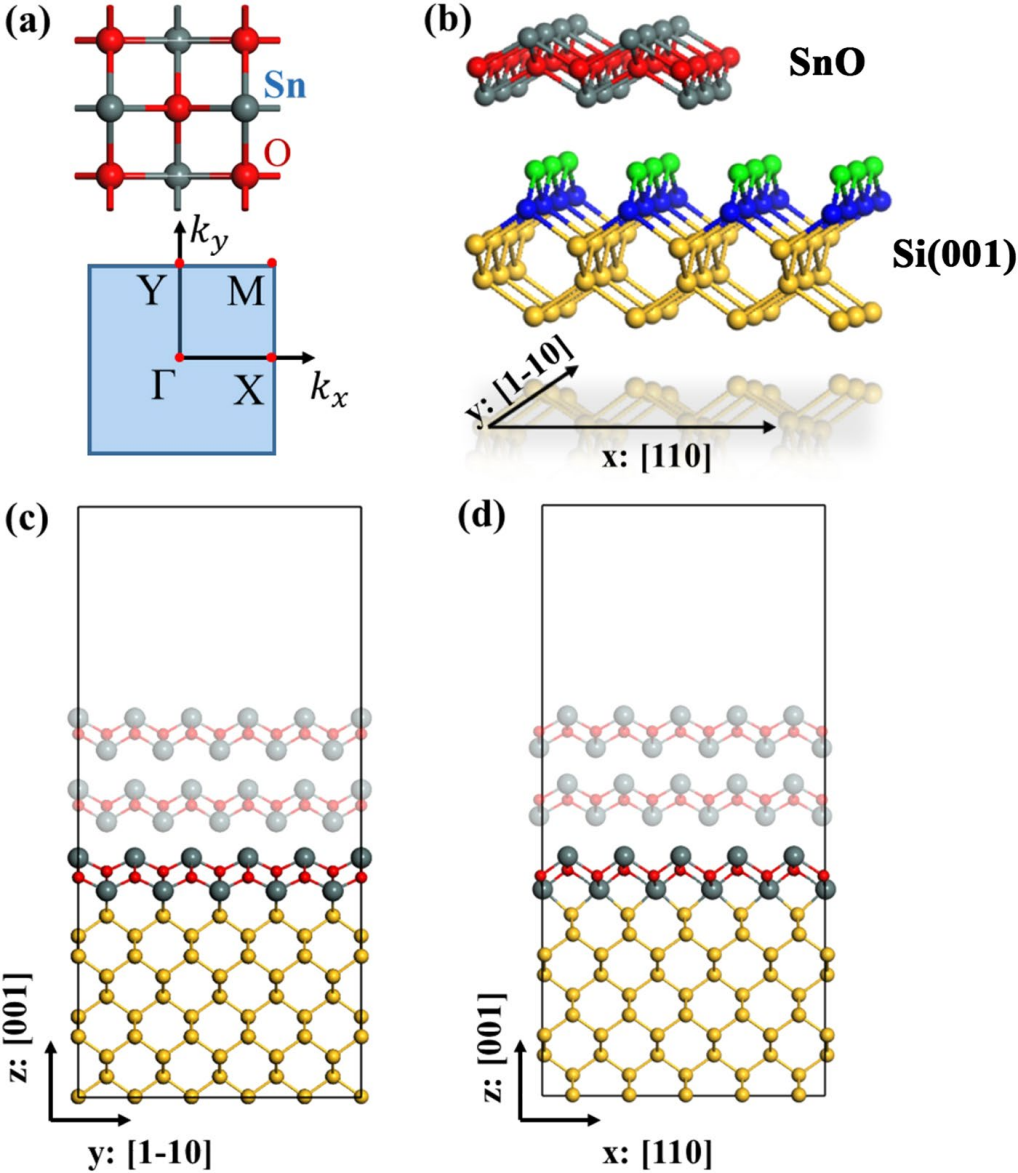
(**a**) The top view of SnO unitcell and two-dimensional Brillouin zone with selected high symmetry points. (**b**) 3D view of SnO layer over clean Si(001)-1 × 1 surface with the 1^st^ and 2^nd^ Si layers shown in green and blue respectively and the rest Si layers in yellow. (**c**) and (**d**) Schematics of most stable SnO/Si(001) structure viewed along x:[110] direction and y:[1–10] direction respectively. It is noted that the interfacial structure viewed along x is different from y due to the two-fold rotational symmetry of Si(001) surface.

Bulk SnO is a layered tetragonal system with the P4/nmm space group symmetry. Layered SnO can be easily produced due to relatively weak interlayer interaction. Indeed, few-layer SnO has been realized in experiment recently^11^. As shown in Fig. 1(a), the SnO monolayer (ML) consists of O square lattice with Sn atoms forming distorted tetrahedra around the O. The calculated lattice parameters of the 2D tetragonal unit cell are a = b = 3.81 Å, in agreement with results of previous calculation^12, 13^ and experimental measured value^14^. Experimentally, few-layer SnO was found to be a p-type semiconductor^11^, which is quite rare since most of the 2D materials discovered to date are of n-type. Our first-principles calculation shows that the free-standing ML-SnO has an indirect bandgap >2.5 eV (Figure S2(a)). The band edges are quite flat, showing a large effective mass. The band edge states are mainly composed of *p* orbitals of Sn atom with a localized character. As the number of layers increases, its bandgap decreases but remains finite (see Figure S2 in Supporting Information).

Currently, 2D SnO has not been realized in free-standing form. For device applications, 2D materials usually need to be supported by a substrate. Being the most commonly used material in semiconducting industry, Si is most desirable substrate. We note that the lattice constant of SnO is very close to that of the Si(001) surface (3.84 Å). The lattice mismatch is small enough to ensure epitaxial growth or deposition of SnO layer onto the clean Si(001) surface. We have investigated several configurations of the SnO/Si(001) heterostructure and define their interfacial energy according to Safdar *et al*.^15, 16^:

1 $${E}_{interfacial}=[{E}_{total}-({E}_{SnO}+{E}_{Si(001)-p2\times 1})]/(2A)$$

where E_total_ is the total energy of the SnO/Si(001) heterostructure, E_SnO_ is the energy of SnO layers, A is the interfacial area, and E_Si(001)−p2 × 1_ is the energy of the clean Si(001) substrate with p2 × 1 reconstruction which is the mostly observed in experiments. We find, first of all, that the relative stability of the interfacial will not be affected by the thickness of SnO due to the fact that ML-SnO will not significantly alter the bonding structure at the SnO/Si interface (see Table S1 in Supporting Information). It is found that the most stable configuration is the structure with the Sn atoms in the lowest SnO layer bonded with the surface Si atoms, as shown in Fig. 1(c) and (d).

**Figure 2:**
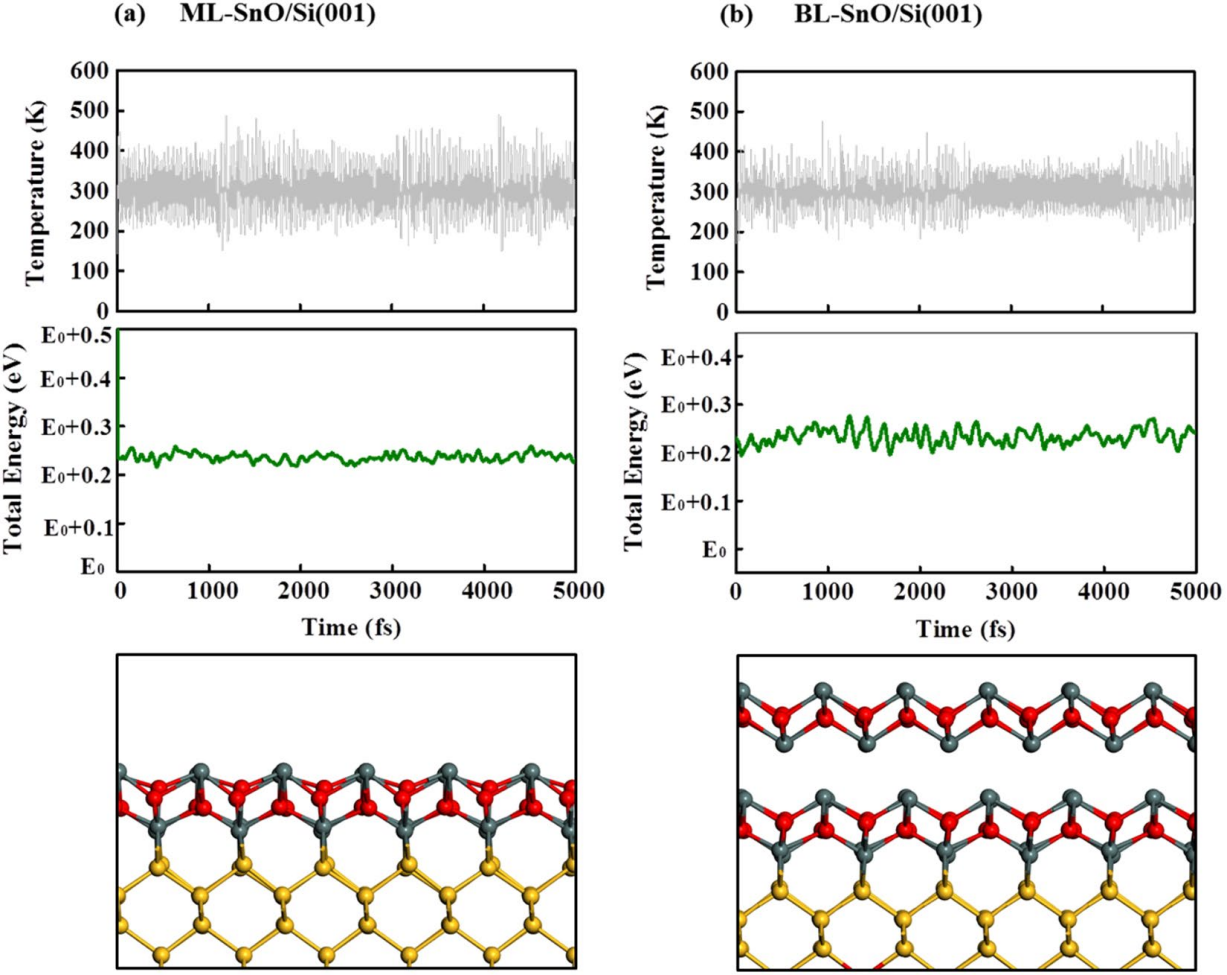
First-principle molecular dynamic calculations at T = 300 K for ML-SnO (**a**) and BL-SnO (**b**) on Si(001) surface. The top panel and the middle panel represent the temperature and total energy respectively as a function of time. The bottom panel shows the snapshot of the corresponding molecular dynamic simulations.

The dynamic stability of this SnO/Si(001) heterostructure is also investigated. We carried out first-principle molecular dynamics simulations at room temperature (T = 300 K) with a time step of 1 fs for both ML-SnO/Si(001) and BL-SnO/Si(001) structures. We can see that the total energy is stable though out our simulation without any tendency of decrease or increase, and, after running 5000 steps, the lattice geometry which can be discerned from both bond angle and the distance between SnO and Si that there is no significant disruptive trend, thus we can determine the lattice geometry is maintained (bottom panel of Fig. 2), suggesting that such systems are stable at room temperature.

**Figure 3:**
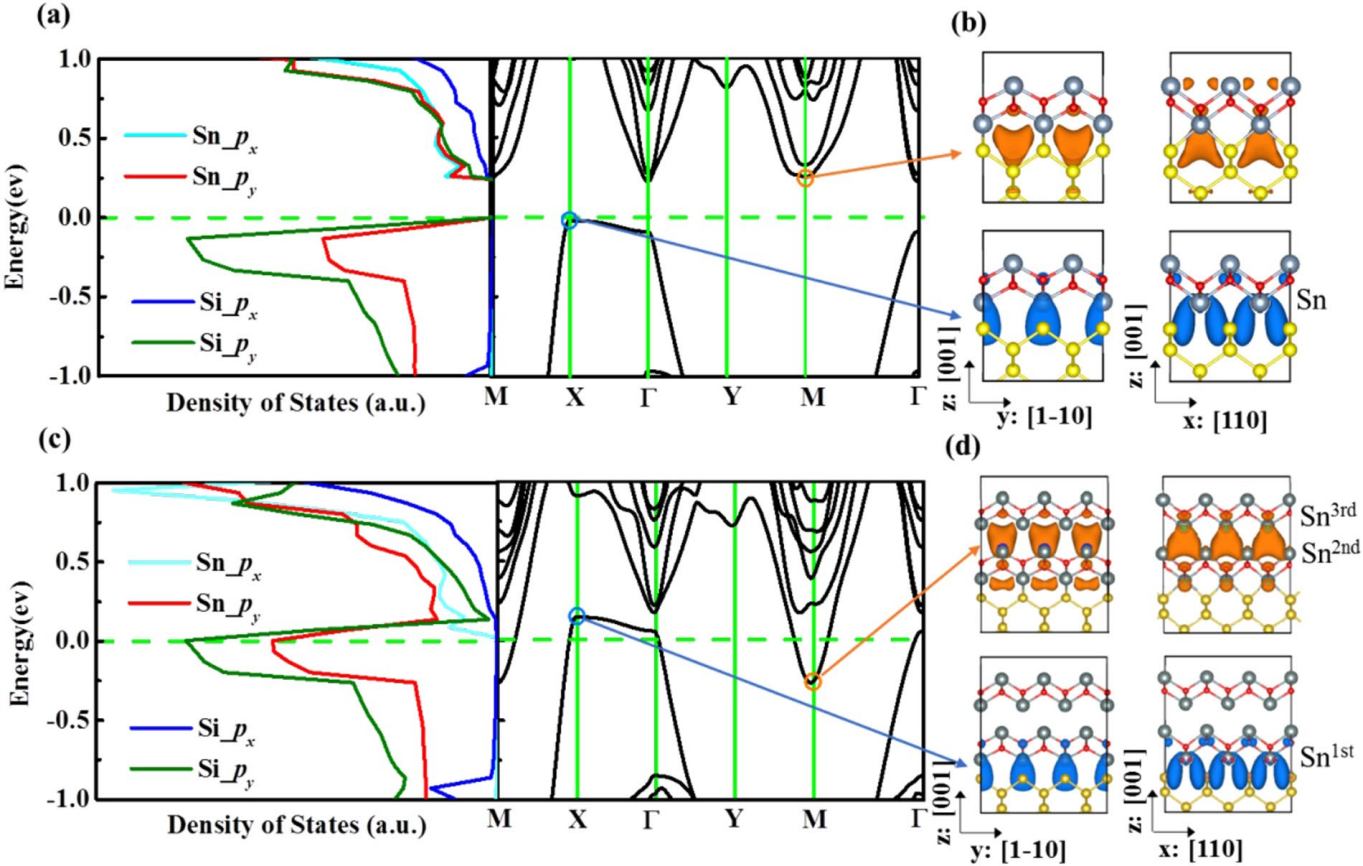
The density of states (DOS) and band structures for ML-SnO/Si(001) (**a**), along with the corresponding charge density at the CBM (M point) and VBM (X point) (**b**). DOS and band structures for BL-SnO/Si(001) (**c**), along with the corresponding charge density at M and X points (**d**).

The interaction with the Si substrate strongly modifies the electronic properties of 2D SnO. Figure 3(a) shows the density of states (DOSs) and the band structure of ML-SnO on Si(001) substrate. One observes that although the system is still semiconducting, its bandgap is decreased to about 0.35 eV. The valence band maximum (VBM) is now located at the X point, while the conduction band minimum (CBM) is at the M point (band edge at the Γ point is also close in energy). Notably, the band structure now exhibits a strong in-plane anisotropy between x:[110] and y:[1–10] directions of Si(001) substrate. This is because the original four-fold rotational symmetry of free-standing ML-SnO is broken by the presence of the Si(001) substrate (only two-fold rotational symmetry remains as shown in Fig. 1(b)). This anisotropy is most pronounced for the VBM states. One observes that the dispersion near VBM is much stronger along XM than along XΓ direction. As shown in Fig. 3(b), the corresponding states near VBM are from the Sn-Si bonds which is extended along the x direction, hence giving the larger dispersion along this direction. Meanwhile, along the y direction, there is little overlap between neighboring Sn-Si bonding states (down-left panel of Fig. 2(b)) due to the highly oriented *p* orbitals between them, resulting in a low dispersion along this direction. These observations are quantified by the anisotropic effective masses, as shown in Table 1. Particularly, the hole effective mass along the x direction is only one third of that of Si and is also much smaller than other 2D materials^17^, suggesting that the transport along this direction can have a very high mobility. The strong anisotropy could also help to confine the carrier flow along one direction to achieve some novel functionality. For CBM, we find that the anisotropy is small because the CBM is mainly composed by Sn-*px* and Sn*-py* orbitals whose distribution are similar along two directions (left panel, Fig. 3(a)). The electron effective mass, although not as small as the hole effective mass, is still comparable with that of Si and MoS_2_ (Table 1). Therefore, the ML-SnO/Si(001) system is a promising system to build high performance 2D electronics, especially in the p-doped regime.

**Table 1:** Effective mass calculated for ML and BL SnO/Si(001) surface, along with the values of MoS_2_ and Si for comparison.

Material	${{\bf{m}}}_{{\bf{h}}}^{{\boldsymbol{\ast }}}/{{\bf{m}}}_{{\bf{0}}}$		${{\bf{m}}}_{{\bf{e}}}^{{\boldsymbol{\ast }}}/{{\bf{m}}}_{{\bf{0}}}$	
	x:[110]	y:[1–10]	x:[110]	y:[1–10]
ML-SnO/Si(001)	−0.122	−15.659	0.833	0.595
BL-SnO/Si(001)	−0.124	−40.860	0.333	0.375
ML-MoS_2_^[1]^	−0.551		0.428	
Si^[2]^	−0.36		0.26	

^[1]^Present calculation.

^[2]^B. Van Zeghbroeck, Principles of Semiconductor Devices.

**Figure 4:**
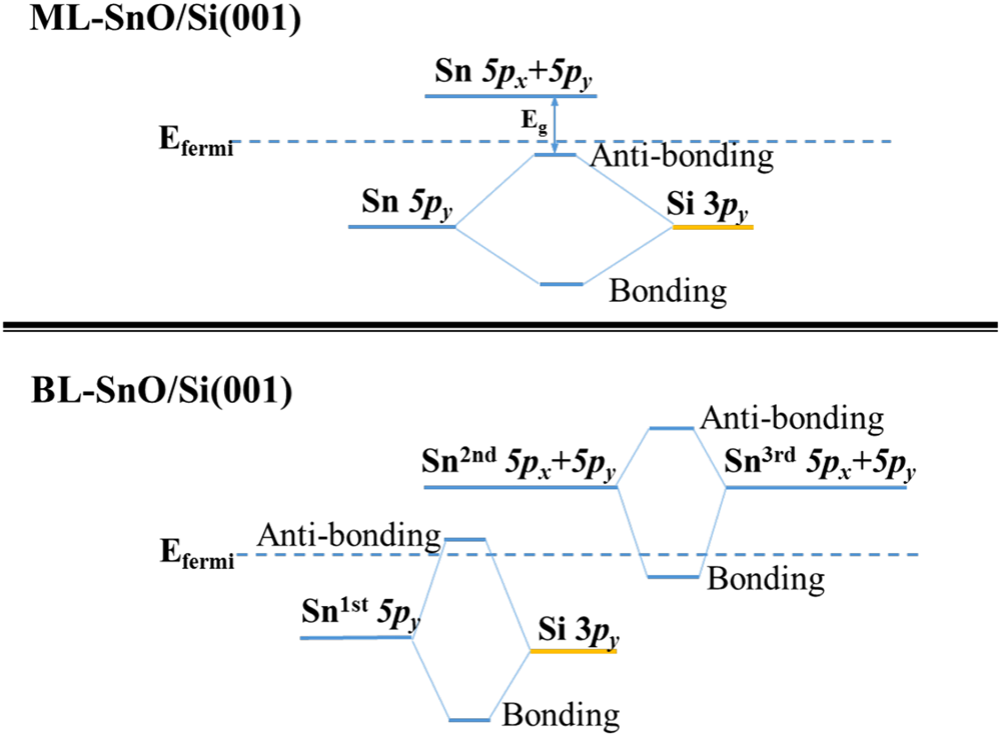
Energy-level evolution diagram around Fermi level associated with the ML-SnO/Si(001) (upper panel) and BL-SnO/Si(001) (lower panel).

Interestingly, when going from monolayer to multilayer SnO on Si(001), the bandgap disappears and the heterostructure becomes metallic. As shown in Fig. 3(c), for the bilayer (BL)-SnO/Si(001) structure, the conduction and the valence bands overlap. Compared with the monolayer case, this gap closing is mainly due to lowering of CBM at the M point, which can be attributed to the lone pair coupling between the two SnO layers. In multilayer SnO, this lone pair coupling is known to be important for the gap narrowing with the increasing number of layers^13^ (see Figure S2). In pure SnO, both VBM and CBM are composed mainly by Sn-5*p* orbital, and the energy level of the occupied Sn-5*p* orbital is very low, leading to a finite bandgap. However, in the presence of the Si substrate, the hybridization between the Si-3*p* orbitals and Sn-5*p* orbitals at the interface forms the new VBM whose energy level is lift up, which is very close to Fermi level in ML-SnO/Si(001) (see schematic energy diagram in the upper panel of Fig. 4). In the case of two or more SnO layers on Si(001), the lone pair coupling between SnO layers (upper panel of Fig. 3(d)) shifts the CBM lower than the VBM at X as illustrated in lower panel of Fig. 4. Thus, the system transforms from semiconductor to semimetal. This metallic character remains with further increase in thickness of SnO (see Figure S3 in Supporting Information). Because the VBM near the X point is mainly from the states near the interface (lower panel of Fig. 3(d)), its energy and dispersion is more or less fixed and not affected by the number of layers of SnO. Additional calculations using hybrid functional at HSE06 level^18^ were also performed to confirm these electronic structures and semiconductor-metal transition (Figure S5).

**Figure 5:**
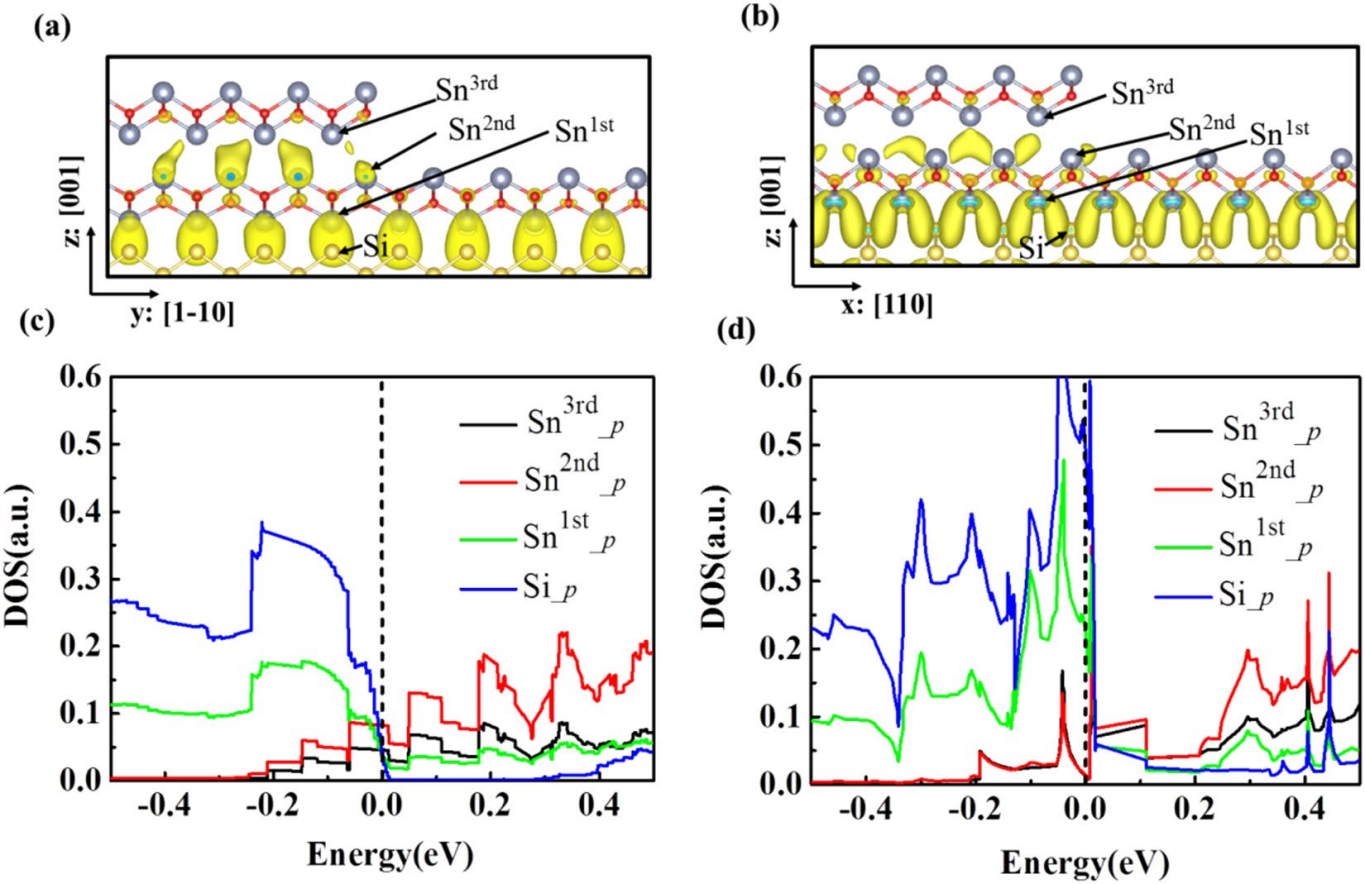
Contour plots of the charge density at the interface of BL and ML SnO/Si(001) along x:[110] direction (**a**) and y:[1–10] direction (**b**) with an “edge up” configuration associated with states in the energy range: *E*_*F*_ − 0.1 < E < *E*_*F*_ + 0.1 eV. (**c**) and (**d**) are the density of states projected on interfacial atoms shown (**a**) and (**b**), respectively.

This layer-dependent semiconductor-metal transition is very important for the construction of a logic devices based on a single 2D material. For example, a lateral junction can be realized between ML-SnO and BL-SnO on a Si(001) substrate. The BL-SnO region naturally provides a metallic electrode, while the ML-SnO regions can be used as the semiconducting channel region. Since the whole device is built on the same Si-flake and implemented on the same material (SnO), the fabrications could be easy and the contact resistance could be minimized. Indeed, there is no serious lattice mismatch problem, and as we have pointed out, the VBM states in both monolayer and bilayer regions are from the same interface states, all contributing to minimize the contact resistance. Figure 5(a) and (b) show the geometrical structure of the contact region between BL-SnO/Si(001) ML-SnO/Si(001) with edge along x:[110] and y:[1–10] directions respectively. We find that the edge structure of the 2nd layer has only minor effects on the interfacial electronic properties (see Figure S4). The electronic transparency of a contact also needs large DOS at Fermi level throughout the interface region. The DOS projection onto interfacial atoms are presented in Fig. 5(c) and (d). It is clear that the DOS at fermi level is large and mainly contributed by the states from the Si-Sn interface and from the interlayer Sn *p*-orbital. This is consistent with the analysis as in Fig. 3(a) and (c). All these factors ensure the efficient transferring of electrons across the lateral junction. We have calculated the I-V curve based on the non-equilibrium Green’s function technique and estimate the contact resistance based on equation:

2 $${R}_{c}={[\frac{\partial V}{\partial J}]}_{V=0}$$

The estimated *R*_c_ is only 1.35 × 10^−12^ Ω × cm^2^, confirming the very low contact resistance of the proposed structure.

**Figure 6:**
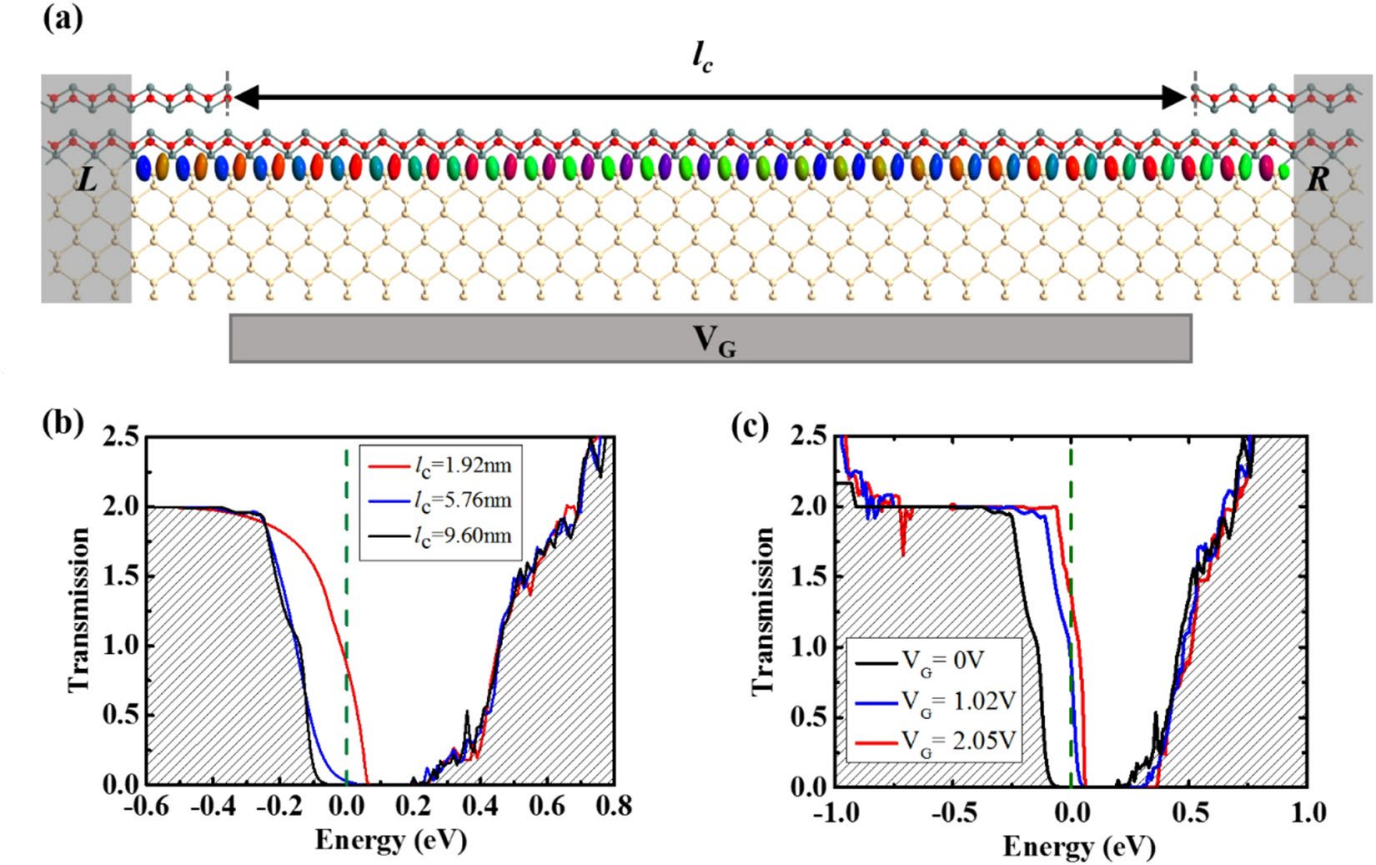
(**a**) Schematic representation of a BL/ML/BL SnO/Si(001) FET with transmission along x:[110] direction. The BL/ML/BL corresponds to the left electrode (L)/channel region (C)/right electrode (R). *l*_*c*_ corresponds to the channel length. Yellow: silicon, red: oxygen and grey: tin. Transmission eigenstate at Fermi level (V_G_ = 1.02 V) is also shown. (**b**) Transmission spectrum with respect to the central length *l*_*c*_. (**c**) Transmission spectrum with *l*_*c*_ = 9.60 nm under different gate voltage.

Now, we consider an FET device based on such a junction. The device is schematically illustrated in Fig. 6(a). It consists of BL/ML/BL regions of SnO/Si(001) with transmission along x:[110] direction. The BL-SnO/Si(001) parts on the two sides are used as source and drain electrodes, and the ML-SnO/Si(001) section is the channel region. The transport characteristic of the device is shown in Fig. 6(b) at zero gate voltage for several channel lengths (*l*_c_). It is clear that the transmission states exist across the Fermi level for the channel length of 1.92 nm. With increasing channel length, the transmission gap is widened. At *l*_c_ = 5.76 nm, there is no transmission peaks around the Fermi level, for which the leakage current would be negligible. This indicates that for the proper operation of the FET, the smallest length of the channel should be larger than 6 nm, and shorter channels would yield leakage currents below the onset.

The controllability by gate voltage is important for the FET performance. Figure 6(c) shows the transmissions spectra of our simulated device (*l*_c_ = 9.6 nm) at different gate voltage. One observes that at V_G_ = 0, i.e. in the off state, there is no transmission at the Fermi level, and the transmission gap is larger than 0.2 eV. When the gate voltage increases above the threshold value ~1.02 V, the electrostatic potential shifts the energy levels of the channel region to cross the Fermi level, and the carriers can move through. The transmission or conductivity is gradually enhanced with further increase of the gate voltage. As is shown in Supporting Information Figure S7, the on-current *I*_on_ is about 450 nA where the off-state leakage current under zero gate voltage is around 5 nA which yield a fairly sufficient ON-OFF ratio ~100. The calculated subthreshold swing (defined as d*V*_gate_/d log *I*)^19^ is approximately 600 mV/decade. Moreover, one notes that the transmission at the interface is mainly contributed by the VBM states located between SnO and Si(001) as shown in Fig. 6(a), because the orbital character of VBM states of tunneling (ML-SnO/Si(001)) is the same as that of electrodes (BL-SnO/Si(001)). The CBM states of ML-SnO/Si(001) (at the SnO/Si(001)) interface, see upper panel of Fig. 3(b)) are different from that of BL-SnO/Si(001) (which are located between the two SnO layers, see upper panel of Fig. 3(d)). Thus, the transmission between the CBM states is low, even under a high gate voltage. The FET device with transmission along y:[1–10] direction is also considered as shown in Figure S6(a), which has the similar transmission character.

## Conclusion

In conclusion, based on first-principle calculations, we propose that 2D SnO layers grown on Si(001) surface is a promising material for future electronics. Our calculations show that ML-SnO/Si(001) has a semiconducting property with strong anisotropy in the effective mass of *p*-type carriers. The hole mass is very small along one direction, indicating a high mobility. The heterostructure becomes metallic with two or more SnO layers on Si(001) surface because of the coupling between SnO layers. This layer-dependent metal–semiconductor dichotomy allows the construction of logical junction based on a single 2D material on Si(001) surface, which naturally minimizes the contact resistance. We propose an FET device based on the structure with the channel length ~6 nm, and excellent performance such as low contact resistance, low leakage current, and easy gate control. Thus, SnO/Si(001) system presents intriguing features that are desired for constructing high performance FETs. The use of Si substrate also makes it easily incorporated into the existing semiconductor technology. Thus, our results suggest 2D SnO/Si(001) as a promising platform for designing the next-generation electronic devices.

## Theoretical Methods

Our first-principles calculations based on the density functional theory (DFT) were carried out using a plane wave basis set and the projector-augmented wave method^20, 21^, as implemented in the VASP code. Kinetic energy cutoff was set above 400 eV for all calculations. For the exchange and correlation functional, the generalized gradient approximation (GGA) in Perdew-Burke-Ernzerhof (PBE)^22^ format was used. In addition, we also did bandstructure calculations using screened hybrid functional of Heyd, Scuseria, and Ernzerhof (HSE06)^18^ to check our main results (see Figure S5), because the hybrid functional approach is better to describe the bandgap. A 24 × 24 × 1 Monkhorst-Pack k-point sampling^23^ is used for the Brillouin zone integration of SnO/Si(001). Si(001) surfaces were modeled by using a slab geometry of ten atomic layers, with a vacuum region of 30 Å in the direction normal to the surface. Test calculations were performed by using larger thickness which gave similar results. Bottom Si layer was terminated by H atoms. During structural optimization, the bottom 4 layers of Si were fixed and other atoms were fully relaxed until the atomic forces were smaller than 0.01 eV/Å. Because of weak interactions between SnO layers, Van der Waals interactions were considered by the vdW-DF level with the optB88 exchange functional (optB88-vdW)^24^.

The transport properties including conductance spectra and eigenchannel of devices built from SnO/Si(001) heterostructure were calculated by using non-equilibrium Green’s function method coupled with DFT as implemented in the Atomistix ToolKit package (ATK 2016.2)^25, 26^. The double-zeta polarized basis set was employed during device simulation. The temperature was set to 300 K and a Monkhorst-Pack K-point mesh of 1 × 24 × 100 yield a good balance between computational time and accuracy in the results. The energy and voltage-resolved transmission function were calculated using:

3 $$T(\varepsilon ,{V}_{{\rm{Bias}}})=Tr[{\hat{G}}_{{\rm{C}}}^{\dagger }{\hat{{\rm{\Gamma }}}}_{{\rm{R}}}{\hat{G}}_{{\rm{C}}}{\hat{{\rm{\Gamma }}}}_{{\rm{L}}}]$$

where ${\hat{G}}_{C}$ is Green’s function of the channel region and ${\hat{{\rm{\Gamma }}}}_{{\rm{L}},{\rm{R}}}$ are the broading function of the left and right electrodes. Both electrodes and the central part are periodic perpendicular to the transport direction. In order to have computationally convenient setup for calculation of broadening functions at the borders, the central region included small adjacent parts of the electrodes as buffer layers.

## Electronic supplementary material

Support Information

## References

[1] Fuechsle M (2010). Spectroscopy of few-electron single-crystal silicon quantum dots. Nat. Nanotechnol..

[2] Fuechsle M (2012). A single-atom transistor. Nat. Nanotechnol..

[3] Kahng A (2010). Scaling: more than moore’s law. IEEE Design & Test of Computers.

[4] Wong H (2002). Beyond the conventional transistor. IBM J. Res. Dev..

[5] Lu Y (2013). A Possible Reaction Pathway to Fabricate a Half‐Metallic Wire on a Silicon Surface. Adv. Funct. Mater..

[6] Geim A, Grigorieva I (2013). Van der waals heterostructures. Nature.

[7] Radisavljevic B, Radenovic A, Brivio J, Giacometti V, Kis A (2011). Single-layer mos2 transistors. Nat. Nanotechnol..

[8] Fuhrer M, Hone J (2013). Measurement of mobility in dual-gated mos_2_ transistors. Nat. Nanotechnol..

[9] Kappera R (2014). Phase-engineered low-resistance contacts for ultrathin mos_2_ transistors. Nat. Mater..

[10] Ghorbani-Asl M, Kuc A, Miró P, Heine T (2016). A single-material logical junction based on 2d crystal pds2. Adv. Mater..

[11] Saji K, Tian K, Snure M, Tiwari A (2016). 2D tin monoxide—an unexplored p‐type van der waals semiconductor: material characteristics and field effect transistors. Adv. Electron. Mater..

[12] Togo A, Oba F, Tanaka I, Tatsumi K (2006). First-principles calculations of native defects in tin monoxide. Phys. Rev. B.

[13] Zhou W, Umezawa N (2015). Band gap engineering of bulk and nanosheet sno: an insight into the interlayer sn–sn lone pair interactions. Phys. Chem. Chem. Phys..

[14] Izumi F (1981). Pattern-fitting structure refinement of tin(II) oxide. J. Solid State Chem..

[15] Nazir S, Behtash M, Cheng J, Luo J, Yang K (2016). Nb and ta layer doping effects on the interfacial energetics and electronic properties of laalo_3_/srtio_3_ heterostructure: first-principles anaylsis. Phys. Chem. Chem. Phys..

[16] Wang Y, Tang W, Cheng J, Nazir S, Yang K (2016). High-mobility two-dimensional electron gas in srgeo_3_- and basno_3_-based perovskite oxide heterostructures: an ab initio study. Phys. Chem. Chem. Phys..

[17] Zhang W, Huang Z, Zhang W, Li Y (2014). Two-dimensional semiconductors with possible high room temperature mobility. Nano Res..

[18] Heyd J, Scuseria G, Ernzerhof M (2003). Hybrid functionals based on a screened coulomb potential. J. Chem. Phys..

[19] Yan Q (2007). Intrinsic current-voltage characteristics of graphene nanoribbon transistors and effect of edge doping. Nano Lett..

[20] Blöchl P (1994). Projector augmented-wave method. Phys. Rev. B.

[21] Kresse G, Joubert D (1999). From ultrasoft pseudopotentials to the projector augmented-wave method. Phys. Rev. B.

[22] Perdew J, Burke K, Ernzerhof M (1996). Generalized gradient approximation made simple. Phys. Rev. Lett..

[23] Monkhorst H, Pack J (1976). Special points for brillouin-zone integrations. Phys. Rev. B.

[24] Klimeš J, Bowler D, Michaelides A (2010). Chemical accuracy for the van der waals density functional. J. Phys.: Condens. Matter.

[25] Taylor J, Guo H, Wang J (2001). Ab initio modeling of open systems: charge transfer, electron conduction, and molecular switching of a c60 device. Phys. Rev. B.

[26] Brandbyge M, Mozos J-L, Ordejón P, Taylor J, Stokbro K (2002). Density-functional method for nonequilibrium electron transport. Phys. Rev. B.

