# Supplementary material for: Layer-dependent semiconductor-metal transition of SnO/Si(001) heterostructure and device application
Source: Sci Rep. 2017 May 31;7:2570. doi: 10.1038/s41598-017-02832-8 (PMC5451440; doi:10.1038/s41598-017-02832-8)
Supplement: Supplementary file 1 — Support Information [file 41598_2017_2832_MOESM1_ESM.pdf]

Layer-dependent semiconductor-metal transition of SnO/Si(001) heterostructure and device application

Chengcheng Xiao[1┼], Fang Wang[1┼], Yao Wang[1], Shengyuan A. Yang[2], Jianzhong Jiang[1], Ming Yang[3], Yunhao Lu[1*], Shijie Wang[3], Yuanping Feng[4],

[1] State Key Laboratory of Silicon Materials, School of Materials Science and Engineering, Zhejiang University, Hangzhou, 310027, China

[2] Research Laboratory for Quantum Materials, Singapore University of Technology and Design, Singapore 487372, Singapore

[3] Institute of Materials Research and Engineering, Agency for Science, Technology and Research (A*-STAR), 2 Fusionopolis Way, Singapore 138634

[4] Department of Physics, National University of Singapore, Singapore 117542, Singapore

┼These two authors have equal contribution

*Correspondence to luyh@zju.edu.cn

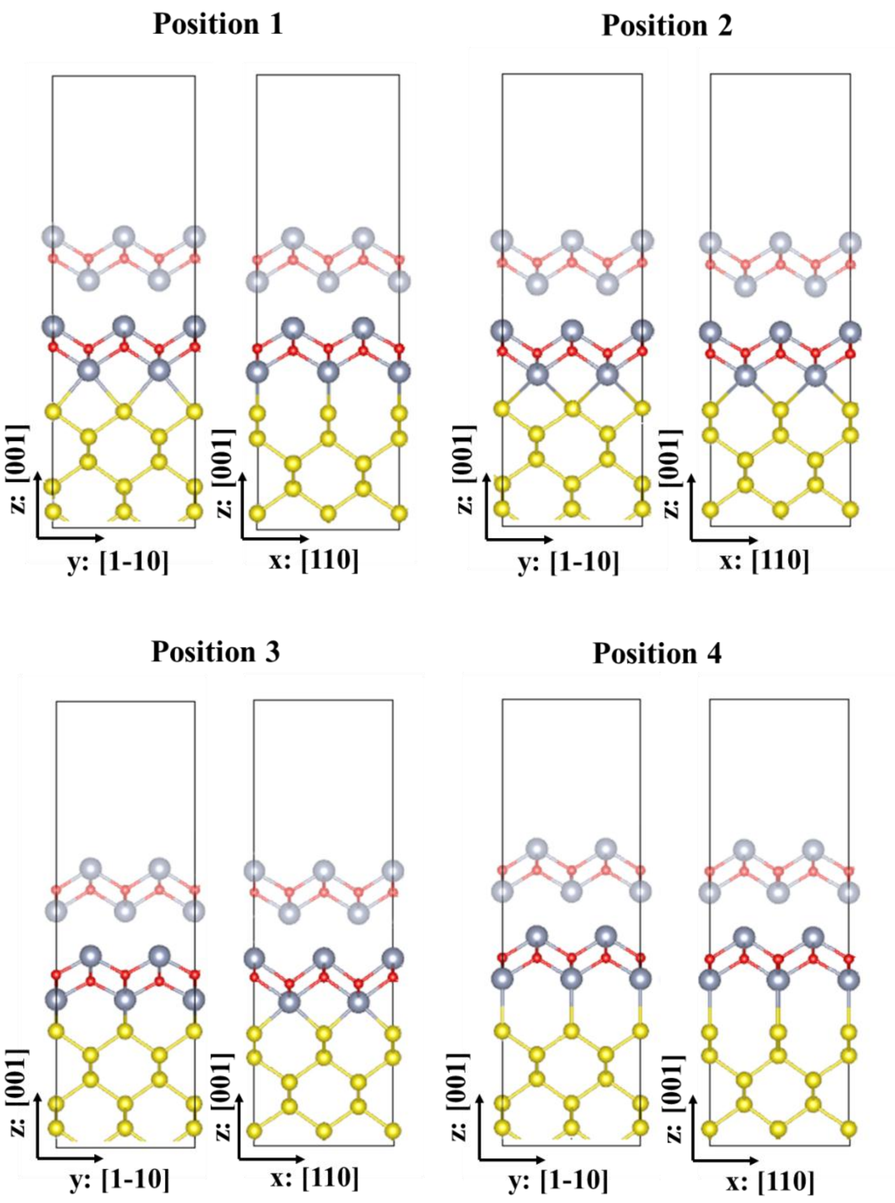

Figure S1: Schema of different SnO/Si (001) structures

Formation Energy(eV/Å^2)	Position 1	Position 2	Position 3	Position 4
SnO 1L	**-0.00651**	**-0.00827**	**-0.0138**	**0.01566**
SnO 2L	**-0.00644**	**-0.00851**	**-0.01472**	**0.01302**

Table S1: Interfacial energies between SnO and Si for different SnO/Si (001)-p2x1 adhesive structures shown in Fig. S1

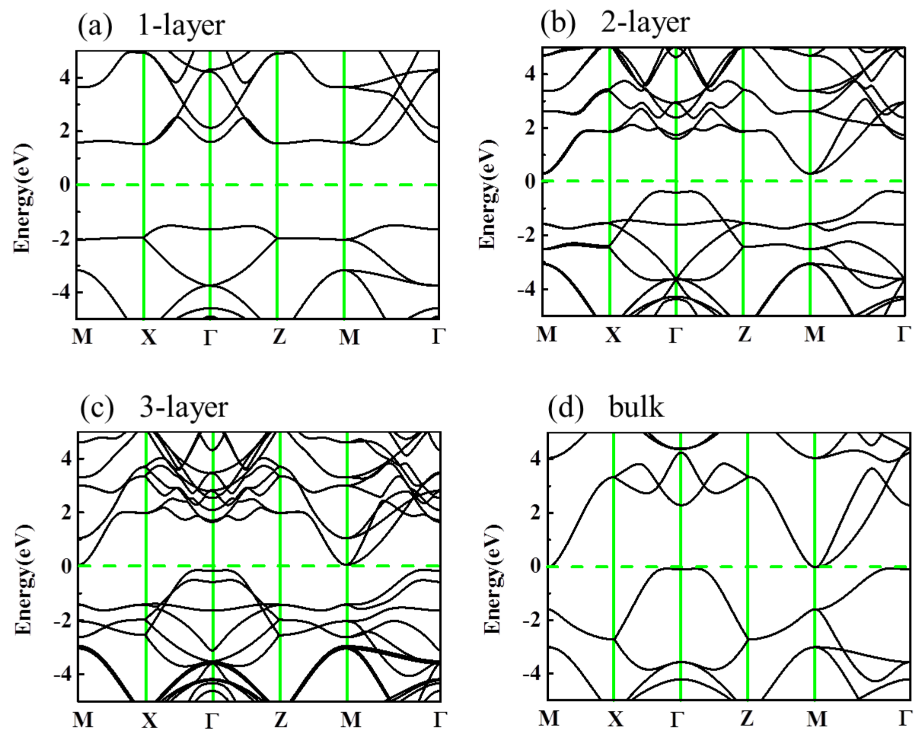

Figure S2 Band structures for 1-3 layer and bulk SnO, respectively.

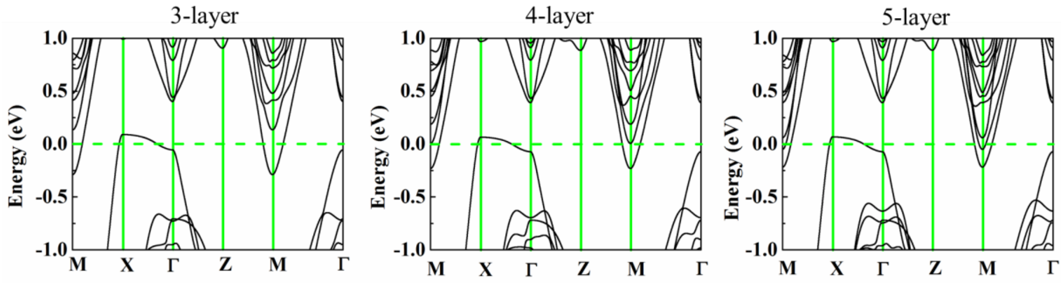

Figure S3 Band structures for 3-5 layers of SnO on the Si(001) surface.

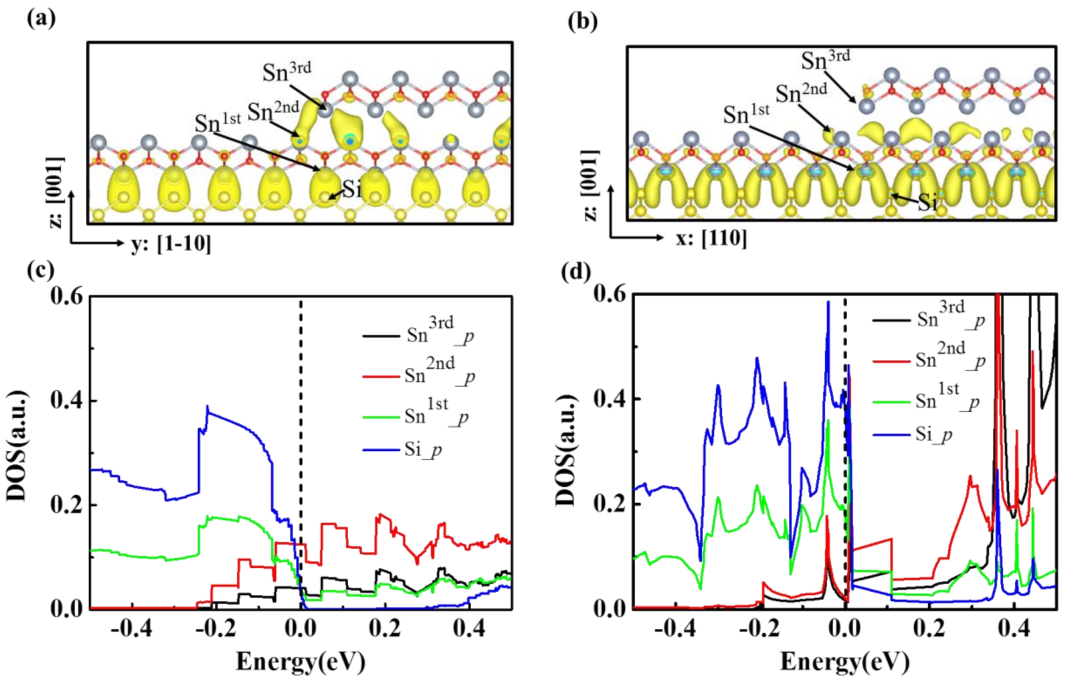

Figure S4 The charge density at the interface of systems with BL and ML SnO/Si(001) along y direction (a) and x direction (b) with an "edge down" configuration within energy range: E_f-0.1~E_f+0.1eV. (c) and (d) are the density of states projected on interfacial atoms corresponding to (a) and (b), respectively.

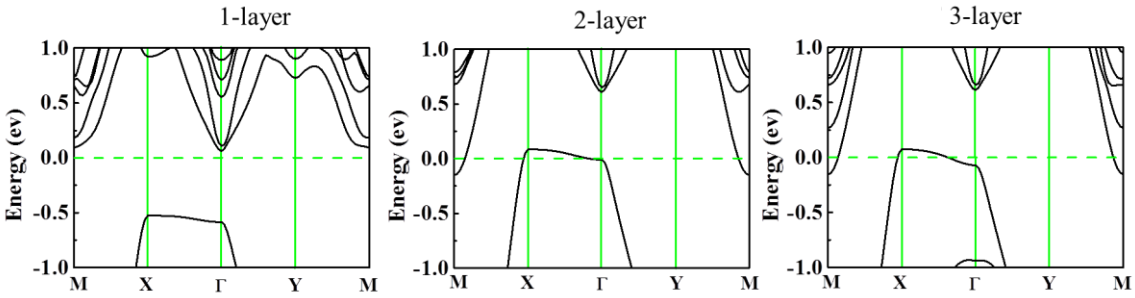

Figure S5 Band structures for 1-3 layers of SnO on Si(001) surface calculated with hybrid functional HSE06.

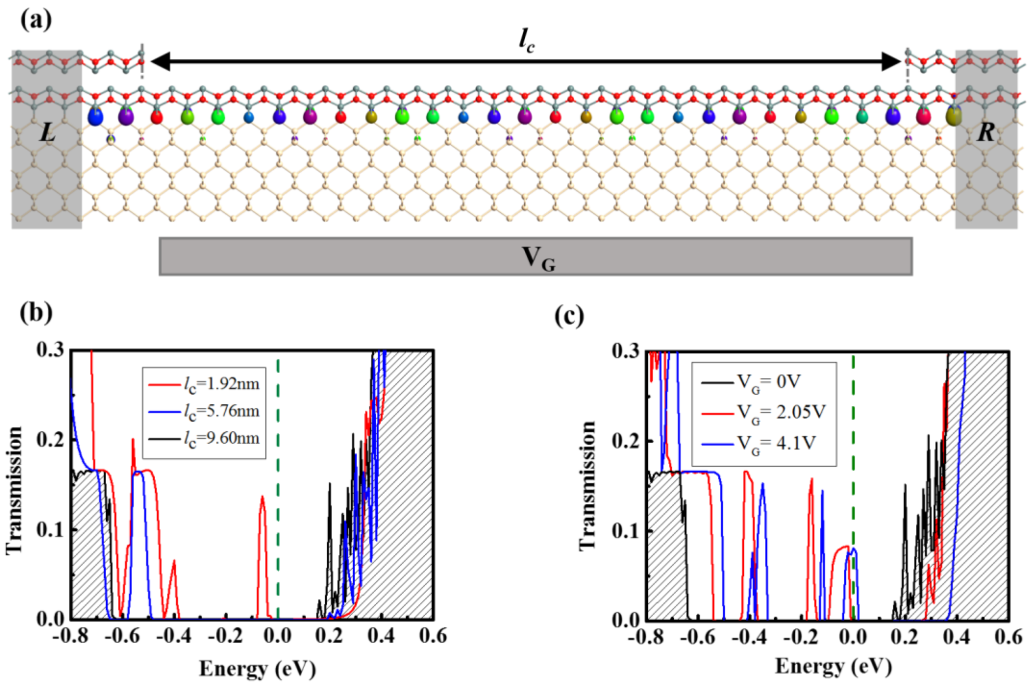

Figure S6 (a) Schematic representation of a BL/ML/BL SnO/Si(001) FET with transmission along y:[1-10] direction. The BL/ML/BL corresponds to the left electrode (L)/channel region (C)/right electrode (R). l_c corresponds to the channel length. Yellow: silicon, red: oxygen and grey: tin. Transmission eigenstate at Fermi level (V_G=2.05V) is also shown. (b) Transmission spectrum with respect to the central length l_c. (c) Transmission spectrum with l_c = 9.60nm under different gate voltage.

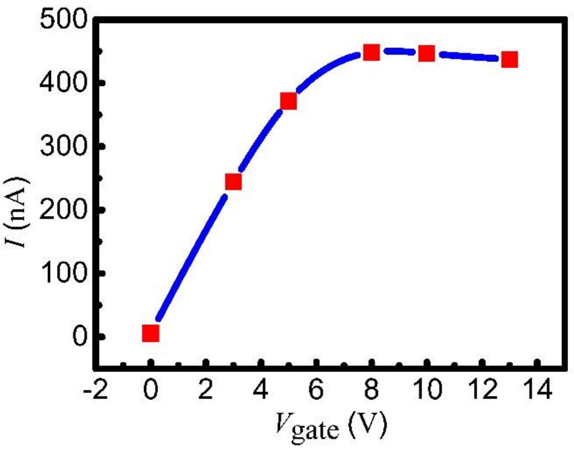

Figure. S7 Current under same bias (10mV) with respect to different gate voltage.
